# Intravenous immunoglobulin therapy in kidney transplant recipients with *de novo* DSA: Results of an observational study

**DOI:** 10.1371/journal.pone.0178572

**Published:** 2017-06-27

**Authors:** Marie Matignon, Caroline Pilon, Morgane Commereuc, Cynthia Grondin, Claire Leibler, Tomek Kofman, Vincent Audard, José Cohen, Florence Canoui-Poitrine, Philippe Grimbert

**Affiliations:** 1 AP-HP, Henri Mondor Hospital, Nephrology and Transplantation Department and CIC Biothérapies 504, Créteil, France; 2 INSERM U955, Team 21, Créteil, France and Paris Est University (UPEC), Créteil, France; 3 AP-HP, Henri Mondor Hospital, CIC Biothérapies 504, Créteil France; 4 AP-HP, Henri Mondor Hospital, Nephrology and Transplantation Department, Créteil, France; 5 INSERM U955, Team 21, Créteil, France; 6 AP-HP, Henri-Mondor Hospital, Public Health Department, Creteil, France; 7 Paris-Est University, UPEC, IMRB-EA 7376 CEpiA unit (Clinical Epidemiology And Ageing), Creteil, France; University of Toledo, UNITED STATES

## Abstract

**Background:**

Approximately 25% of kidney transplant recipients develop *de novo* anti-HLA donor-specific antibodies (*dn*DSA) leading to acute antibody-mediated rejection (ABMR) in 30% of patients. Preemptive therapeutic strategies are not available.

**Methods:**

We conducted a prospective observational study including 11 kidney transplant recipients. Inclusion criteria were *dn*DSA occurring within the first year after transplant and normal allograft biopsy. All patients were treated with high-dose IVIG (2 g/kg 0, 1 and 2 months post-*dn*DSA). The primary efficacy outcome was incidence of clinical and subclinical acute ABMR within 12 months after *dn*DSA detection as compared to a historical control group (IVIG-).

**Results:**

Acute ABMR occurred in 2 or 11 patients in the IVIG+ group and in 1 of 9 patients in the IVIG- group. IVIG treatment did not affect either class I or class II DSA, as observed at the end of the follow-up. IVIG treatment significantly decreased FcγRIIA mRNA expression in circulating leukocytes, but did not affect the expression of any other markers of B cell activation.

**Conclusions:**

In this first pilot study including kidney allograft recipients with early *dn*DSA, preemptive treatment with high-dose IVIG alone did not prevent acute ABMR and had minimal effects on DSA outcome and B cell phenotype.

## Introduction

Humoral allo-immune responses mediated by donor-specific antibodies (DSA) is now well recognized as a detrimental determinant of graft outcome after solid organ transplantation [1]. DSA identified before transplantation in patients already sensitized are associated with both acute and chronic antibody-mediated rejection and poorer graft survival [2–6]. Alternatively, *de novo* DSAs (*dn*DSAs) emerge after transplantation in 13%–27% of previously non-sensitized recipients when detected with current highly sensitive single-antigen flow beads (SAFB) assays [7–10]. They usually appear during the first year and most *dn*DSA are anti–class II [7–10]. C1q binding ability of DSA and DSA subclass has recently been described to be independently associated with allograft failure [7]. African American race, combined kidney and pancreas transplant, and increased number of HLA mismatches between donor and recipient are clinical risk factors for the development of *dn*DSA [8]. Incidence of acute rejection in kidney allograft recipients with *dn*DSA can reach 50%, with up to 30% subclinical acute rejection [8–10]. Among those 50% are acute antibody-mediated rejections (ABMR) [8–10], which constitute the principal risk factor of graft loss [8, 10]. *dn*DSA are also associated with subclinical histologic lesions, which are an important determinant of graft survival [11, 12]. Thus, current recommendations are to screen for the development of *dn*DSA at 3 months, one year, and once a year after kidney transplant [13]. Although the pathogenicity of *dn*DSA has been clearly established, no preemptive strategy has been proposed so far.

We conducted the first prospective pilot study including 11 kidney transplant recipients with *dn*DSA appearing in the first year after transplant, and without histological evidence of ABMR at the time of *dn*DSA discovery. We analyzed the effect of intravenous immunoglogulin (IVIG) on the incidence of both clinical and subclinical ABMR, DSA outcome and B cell phenotype, comparing the outcomes to those of a historical control group.

## Material and methods

### Patients

Between January 2014 and December 2014, we conducted a prospective pilot study including 11 kidney allograft patients. Inclusion criteria were *dn*DSA occurring within the first year after transplant, and normal allograft biopsy at the time of the DSA discovery (biopsy #1). All patients were treated with high-dose IVIG (2 g/kg at 0, 1 and 2 months). A second biopsy (biopsy #2) was performed 9–12 months after DSA detection. An allograft biopsy was also performed at the time of any clinical event (acute renal failure and/or proteinuria). The control historical group included 9 kidney allograft recipients with *dn*DSA occurring in the first year after transplant, normal allograft biopsy at the time of *dn*DSA detection, and a follow-up biopsy 9–12 months after the *dn*DSA. This study was reviewed and approved by the Paris-4 institutional review board (CPP-APHP_2021). All patients were informed with information letter and gave verbal consent to be included in our study.

### Outcomes

The primary efficacy outcome was the incidence of acute ABMR (clinical and subclinical) within 12 months after the discovery of *dn*DSA. Secondary outcomes were microvascular lesions without acute ABMR and estimated GFR (eGFR; MDRD formula [14]) at the time of biopsy #2, incidence of acute T-cell mediated rejection (TCMR), patient and kidney allograft survival, and changes in *dn*DSA characteristics and B-cell phenotype between the 1^st^ and 2^nd^ biopsies.

### Anti-HLA antibody screening

Low resolution DNA typing was performed in donors and high resolution typing in recipients. All patients included in the study were DSA-negative (using high resolution Luminex single antigen beads (SAB) assay technology), as determined at the time of kidney transplant, and confirmed with 1 or more additional serum samples obtained before transplant.

Serum from stable kidney allograft recipients were screened for the presence of circulating *dn*DSA directed against donor’s HLA-A, HLA-B, HLA-Cw, HLA-DR or HLA-DQ antigens, using high resolution Luminex SAB assay technology, at 3 and 12 months after transplant. A mean baseline normalized MFI greater than 1000 was classified as a positive result. For each serum sample, the number of DSA subclasses, the sum of MFIs for each subclass, and the maximum MFI were analyzed.

### Histologic analysis

Kidney allograft biopsies were analyzed using the Banff’13 updated classification [15].

### B lymphocyte analysis

Peripheral blood mononuclear cells (PBMC) were isolated by centrifugation through Ficoll-Hypaque of blood samples obtained at the time of allograft biopsy, and stored at—80°C until analyzed. After thawing, 0.5–1 X 10^6^ PBMC were incubated with various antibody combinations for 30 min at 4°C. The fluorochrome-conjugated monoclonal antibodies used were anti-CD19 V500, anti-CD3 V450, anti-CD56 PE, anti-CD14 PE-Cy7, anti-CD45 APC, anti-CD38 PE-Cy7 from BD Biosciences, anti-CD24 APC from Miltenyi Biotec and anti-IgD FITC and anti-CD27 PE from Beckman Coulter. Samples were analyzed with Canto II cytometer (BD Biosciences) and the data were processed using FlowJo software (Tree Star, Ashland, USA).

### qPCR analysis

RNA was isolated from blood collected at the time of kidney allograft biopsy, using PAXgene blood RNA tubes (PreAnalytix, Qiagen) according to the manufacturer’s instructions. Real-time quantitative PCR was performed using commercially available primer and probe sets (Applied Biosystems): HPRT: Hs99999909_m1, CD19: Hs00174333_m1, CD32a: Hs00234969_m1, CD32b: Hs00269610_m1, BANK1: Hs00215678_m1, BAFF-R: Hs00606874_g1, BAFF: Hs00198106_m1, APRIL: Hs00601664_g1, TACI: Hs00963364_m1, BCMA: Hs03045080_m1. All samples were tested in duplicate in 96-well plates with the 7900HT fast real-time PCR system (Applied Biosystems, Foster City, CA, USA). HPRT mRNA was used as an endogenous control to normalize RNA amounts. The mRNA level in samples was expressed relative to a reference group (PBMC) using the 2^-ΔΔct^ method.

### Statistical analysis

Quantitative variables are presented as mean (±SD), or as median (first quartile-third quartile or Q1-Q3), and were compared with unpaired t test or non-parametric Mann Whitney test, based on the distribution of the variable. Qualitative values are expressed as % incidence, and were compared with Fisher test or Chi-square test. A paired test was used to compare allograft histology at biopsy #2 vs. biopsy #1. Flow cytometric and qPCR data were analyzed with the non-parametric Mann-Whitney test. A *P*-value < 0.05 was considered statistically significant. All analyses were performed using GraphPad Prism version 5.01 for Windows (GraphPad software, Inc., San Diego, CA).

## Results

Eleven patients receiving kidney allografts between January 2014 and December 2014, and fulfilling inclusion criteria described above, were treated with high-dose IVIG (IVIG+ group). The historical control group included 9 patients (IVIG- group). As depicted in Table 1, demographic and transplant characteristics were similar in both groups.

**Table 1:** Clinical characteristics of treatment groups.

Variables	Whole cohort N = 20	IVIG+ N = 11	IVIG- N = 9	P
**At the time of transplant**				
**Recipient**				
**Female sex, N (%)**	8 (40)	5 (45)	3 (33)	0.50
**Age, years, mean (SD), range**	47 (± 15) (22–75)	45 (± 17) (28–75)	50 (± 15) (22–63)	0.54
**Initial nephropathy, N (%)**				
Glomerular	8 (40)	6 (55)	2 (28)	0.31
Genetic	1 (5)	1 (9)	0 (0)	
Other	11 (55)	4 (45)	6 (66)	
**Dialysis, N (%)**	16 (80)	10 (90)	6 (66)	1.00
Time, months, median (Q1-Q3)	49 (33–57)	51 (40–61)	42 (21–89)	0.67
**Donor**				
**Deceased, N (%)**	17 (85)	9 (81)	6 (66)	1.00
**Female sex, N (%)**	11 (55)	6 (55)	5 (55)	1.00
**Age, years, mean (SD)**	49 (±16)	52 (±16)	44 (±17)	0.34
**eGFR (ml/min/1,73m2), mean (SD)**	89 (±36)	81 (±40)	103 (±27)	0.21
**Proteinuria (g/l), median (Q1-Q3)**	0.14 (0.07–0.47)	0.14 (0.11–0.68)	0.06 (0.04–0.25)	0.04
**Cold ischemia time, hours, mean (SD)**	18 (±7)	18 (±5)	18 (±11)	0.90
**HLA mismatch**				
class I, mean (SD)	3 (±1)	3 (±1)	3 (±1)	1.00
class II, mean (SD)	2 (±1)	2 (±1)	3 (±1)	0.23
**Anti-HLA antibodies, N (%)**	8 (40)	5 (45)	3 (33)	0.32
**Donor specific antibodies, N (%)**	0 (0)	0 (0)	0 (0)	.
**Immunosuppression**				
**Induction, N (%)**	20 (100)	11 (100)	9 (100)	1.00
Anti-thymocyte globulin, N (%)	11 (55)	7 (64)	4 (43)	0.63
Interleukin-2 receptor antibody, N (%)	9 (45)	4 (36)	5 (57)	
**Post-transplant**				
**Maintenance therapy**				
Steroids, N (%)	20 (100)	11 (100)	9 (100)	0.65
Calcineurin inhibitors, N (%)	20 (100)	11 (100)	9 (100)	
Cyclosporine, N (%)	9 (45)	4 (36)	5 (55)	
Tacrolimus, N (%)	11 (55)	7 (64)	4 (44)	
Mycophenolate mofetil, N (%)	20 (100)	11 (100)	9(100)	
**Infectious diseases, N (%)**				
CMV viremia, N (%)	2 (10)	2 (18)	0 (0)	0.49
Before IVIG, N (%)	.	1 (50)	.	
BK virus viremia, N (%)	2 (10)	1 (9)	1 (11)	1.00
Before IVIG, N (%)	.	1 (100)	.	
**Last follow-up**				
eGFR, ml/min/1.73m^2^, mean (SD)	56 (±22)	58 (±25)	53 (±20)	0.60
Allograft loss, N (%)	0 (0)	0 (0)	0 (0)	.
Death, N (%)	0 (0)	0 (0)	0 (0)	.

*Dn*DSA were detected 3 (range 2–5) months after transplant in IVIG+ group and 5 (range 3–7) months after transplant in IVIG—group (P = 0.25). The total number of class I and class II *dn*DSA, and the maximum and sum of MFI for each HLA subclass were similar in both groups (Table 2). One patient presented with class I and class II *dn*DSA. No patient presented with acute renal failure or significant proteinuria. All patients fulfilled histological inclusion criteria (biopsy #1) (Table 2). Two patients in the IVIG+ group had mild acute T-cell mediated histological lesions without acute TCMR. None of them was treated with steroids. Mean eGFR was 63 (±20) ml/min/1.73m^2^ in IVIG+ group and 53 (±15) ml/min/1.73m^2^ in IVIG—group (P = 0.30).

**Table 2:** At the time of *de novo* DSA (biopsy #1).

Variables	IVIG+ N = 11	IVIG- N = 9	P
**Delay from transplant, months, median (Q1-Q3)**	3 (2–5)	5 (3–7)	0.25
**Class I DSA**			
N (%)	7 (64)	5 (55)	1.00
Number, median (Q1-Q3)	1 (1–2)	1 (0–1)	0.39
MFI max, median (Q1-Q3)	2340 (1343–3354)	2049 (1375–2546)	0.78
MFI sum, median (Q1-Q3)	3410 (1773–4712)	2482 (1528–3204)	0.32
**Class II DSA**			
N (%)	5 (45)	3 (33)	1.00
Number, median (Q1-Q3)	0 (0–1)	0 (0–1)	0.72
MFI max, median (Q1-Q3)	1672 (1225–5526)	1583 (1000–5896)	0.78
MFI sum, median (Q1-Q3)	1672 (1225–6102)	1583 (1000–5896)	0.78
**Histology**			
Glomerulitis, N (%)	0 (0)	0 (0)	.
Peri-tubular capillaritis, N (%)	0 (0)	0 (0)	.
Interstitial inflammation, N (%)	1 (9)	0 (0)	1.00
Grade, 1/2/3	1/0/0	.	.
Tubulitis, N(%)	2 (18)	0 (0)	0.49
Grade, 1/2/3	2/0/0	0 (0)	.
Chronic glomerulopathy, N (%)	0 (0)	0 (0)	.
Interstitial fibrosis, N (%)	5 (45)	3 (43)	1.00
Grade, 1/2/3	3/1/1	1/2/0	.
Tubular atrophy, N (%)	4 (36)	3 (43)	1.00
Grade, 1/2/3	3/0/1	2/1/0	.
Vascular			
cv, N (%)	3 (27)	2 (28)	1.00
Grade, 1/2/3	2/1/0	2/0/0	.
ah, N (%)	4 (36)	4 (57)	0.63
Grade, 1/2/3	3/1/0	3/1/0	.
C4d+, N (%)	0 (0)	0 (0)	.
**eGFR (ml/min/1,73m2), mean (SD)**	63 (±20)	53 (±15)	0.30
**Proteinuria (g/L), mean (SD)**	0.07 (±0.03)	0.09 (±0.06)	0.60

**Fig 1:**
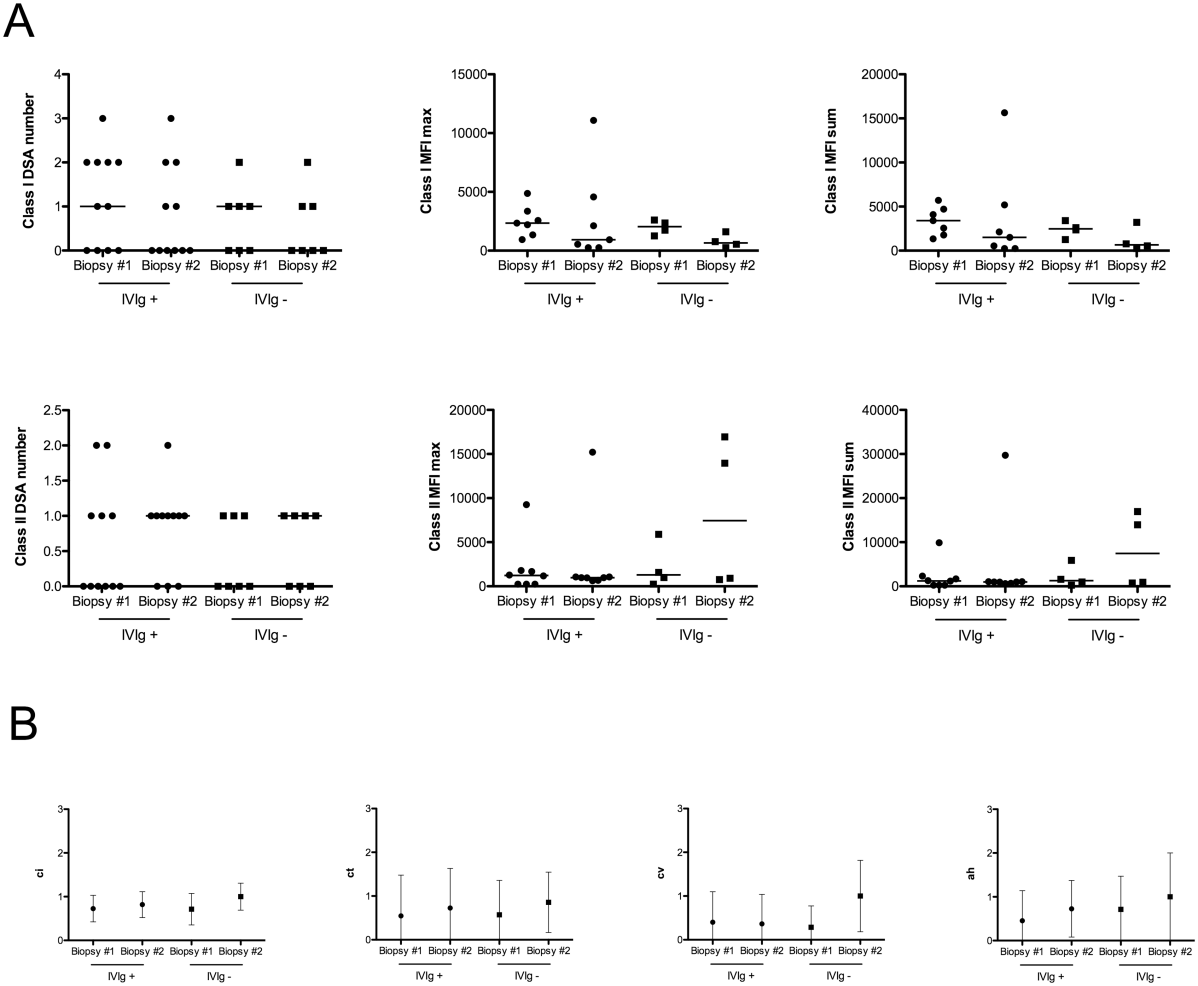
Evolution of the DSA characteristics and lesion histology from the time of *dn*DSA detection (biopsy #1) to the time of the second biopsy (biopsy #2). 1.A: Evolution of class I and class II DSA characteristics (number, MFI max and MFI sum). The evolution of all DSA characteristics was similar in the IVIG+ and the IVIG- group. DSA characteristics were also similar in the IVIG+ *vs*, IVIG- group, at both time points tested. 1.B: Evolution of chronic histologic lesions (ci, ct, cv and ah) as defined in Banff’13 updated classification. No histological differences were found between biopsy #1 and #2, in either the IVIG+ or IVIG- groups. The IVIG+ and control groups were also similar at both time points.

Biopsy #2 was performed 10.5 (9–12) months after *dn*DSA detection in IVIG+ group, and 10.8 (9–12) months in IVIG- group (P = 0.99). Two patients in the IVIG+ group developed acute ABMR 4 (g1, cpt1 and C4d positive) and 7 (g2) months after *dn*DSA detection, whereas one patient in IVIG- group presented with mixed acute rejection (i1, t1, cpt2, C4d negative) 14 months after *dn*DSA detection. At the time of acute rejection, the three patients were treated with steroids pulses, plasmapheresis, Rituximab and four monthly courses of high dose IVIG. The histologic characteristics of the follow-up biopsy #2 were not different between groups. Evolution of chronic (Fig 1B) and acute (data not shown) lesions between allograft biopsy #1 and #2 was similar in both groups. Estimated GFR was 57 (±18) and 54 (±17) ml/min/1.73m^2^ in the IGIV+ and IGIV- groups, respectively (P = 0.72). Proteinuria levels were similar in both groups (P = 0.46).

Class I and class II DSA characteristics and outcomes at the end of follow-up are depicted in S1 Table. Class I and class II DSA number, maximum MFI and sum of MFI were not different in the IVIG+ *vs*. the IVIG- group. Moreover, evolution of class I and class II DSA characteristics, between biopsy #1 and #2, were similar (Fig 1A).

We also analyzed blood leukocyte populations and phenotypes at the time of biopsy #1 (*dn*DSA discovery or baseline) and biopsy #2 (follow-up). In both groups, the proportion of T cells (CD3^+^), monocytes (CD14^+^), NK cells (CD56^+^CD3^**-**^) and B cells (CD19^+^) were unchanged at follow-up *vs*. baseline (Table 3). The proportion of CD24^hi^CD38^hi^, CD24^hi^CD38^-^ cells, and CD24^int^CD38^int^ were also similar at follow-up *vs*. baseline, in both the IVIG+ and IVIG- groups (Table 3). Thus, IVIG treatment did not affect B cell phenotype or increase transitional B cells in kidney transplant recipients with *dn*DSA.

**Table 3:** Blood leukocyte phenotypes and gene expression before and after IVIG treatment.

	IVIg+			IVIg-		
**Populations**	**First** (**%**. [95%IC]) N = 3	**Follow-up** (**%**. [95%IC]) N = 6	p-value	**First** (**%**. [95%IC]) N = 4	**Follow-up** (**%**. [95%IC]) N = 3	p-value
**T cells (CD3**^**+**^**)**	**39.0** [25.7–42.3]	**38.9** [25.1–53.0]	0.714	**54.1** [30.7–84.3]	**15.5** [13.7–56.1]	0.229
**Monocytes (CD14**^**+**^**)**	**41.4** [21.5–41.5]	**33.2** [25.4–50.0]	0.905	**19.8** [5.1–34.8]	**58.5** [23.3–59.5]	0.229
**NK cells (CD56**^**+**^**CD3**^**-**^**)**	**13.3** [4.0–19.7]	**8.5** [4.2–13.9]	0.714	**6.8** [1.4–13.0]	**6.1** [1.4–11.2]	0.857
**B cells (CD19**^**+**^**)**	**7.7** [2.7–16.9]	**6.4** [2.9–9.2]	0.487	**8.6** [3.5–13.3]	**9.4** [5.0–16.3]	0.857
** CD24**^**hi**^**CD38**^**hi**^	**1.74** [1.46–1.74]	**1.94** [1.5–4.48]	0.237	**2.39** [1.25–10.7]	**7.1** [1.64–12.20]	0.857
** CD24**^**int**^**CD38**^**int**^	**39.5** [38.2–44.7]	**37.2** [29.9–53.0]	0.548	**44.8** [40.9–54.8]	**24.6** [23.6–34.5]	0.057
** CD24**^**+**^**CD38**^**-**^	**19.7** [12.4–37.3]	**20.4** [15.5–31.4]	0.795	**18.5** [11.9–19.1]	**21.9** [13.1–40.2]	0.400
** CD27**^**+**^	**33.4** [15.1–57.0]	**30.7** [20.3–44.4]	0.905	**20.3** [15.2–25.6]	**40.9** [27.7–42.9]	0.057
** IgD**^**+**^**CD27**^**-**^	**54.2** [34.3–72.3]	**51.2** [41.7–67.1]	0.795	**58.4** [49.6–73.9]	**44.7** [31.6–59.4]	0.229
**Genes**	**First** Fold increase [95%IC] N = 5	**Follow-up** Fold Increase [95%IC] N = 9	p-value	**First** Fold Increase [95%IC] N = 4	**Follow-up** Fold Increase [95%IC] N = 5	p-value
**CD19**	**6.44** [3.99–18.62]	**3.35** [0.78–4.72]	0.076	**3.45** [1.84–4.99]	**3.53** [2.09–5.32]	1.000
**CD32a**	**23.33** [13.53–37.25]	**11.66** [7.52–16.65]	0.029	**12.6** [8.46–28.08]	**14.82** [5.24–15.48]	0.905
**CD32b**	**8.93** [3.34–14.11]	**6.08** [2.99–14.62]	0.797	**7.38** [4.59–8.68]	**5.37** [2.96–12.06]	0.905
**BANK1**	**1.02** [0.33–3.25]	**0.88** [0.44–1.47]	0.898	**1.22** [0.86–1.60]	**1.27** [0.66–1.75]	0.905
**BAFFR**	**0.21** [0.08–1.38]	**0.25** [0.03–0.46]	0.413	**0.17** [0.12–0.46]	**0.23** [0.14–0.79]	0.413
**BAFF**	**36.1** [27.58–86.7]	**18.37** [8.41–42.07]	0.083	**22.44** [14.93–33.04]	**26.98** [11.08–42.16]	0.905
**BCMA**	**0.014** [0.003–0.152]	**0.029** [0.003–0.075]	1.000	**0.035** [0.015–0.037]	**0.041** [0.012–0.081]	0.413
**TACI**	**0.905** [0.273–2.508]	**0.669** [0.143–1.075]	0.606	**0.575** [0.516–0.753]	**1.189** [0.247–2.195]	0.730
**IL-10**	**0.383** [0.280–1.568]	**0.328** [0.241–0.514]	0.364	**0.260** [0.128–1.063]	**0.239** [0.147–0.330]	0.730

We also determined whether IVIG treatment affected the expression of molecules involved in B cell function, including those associated with: inhibition (CD32 isoforms and the B-cell scaffold protein ankyrin repeats 1 BANK1), survival and differentiation (the A proliferating inducing ligand APRIL), and maturation (BCMA), as well as the B-cell activating factor (BAFF) and BAFF receptors (BAFF-R, the transmembrane activator and CAML interactor TACI). The transcript level of each factor in total blood obtained at the time of biopsy #2 was compared to the corresponding levels at the time of biopsy #1. Only CD32a mRNA levels significantly decreased in the IVIG+ group with *dn*DSA (P = 0.03), while remaining stable in the IVIG- group (Table 3).

## Discussion

We report here the results of the first prospective pilot study designed to determine the effects of high-dose IVIG alone on clinical, histological and immunological outcomes in renal transplant recipients with *dn*DSA appearing during the first year after transplant. In our study, we did not identify beneficial effects of IVIG in terms of preventing acute ABMR, or altering DSA outcome or B-lymphocyte phenotype, with the exception of a significant decrease in CD32a mRNA expression in circulating leukocytes of IVIG-treated patients, which was not seen in historical control patients.

Acute and chronic ABMR play an increasingly critical role in kidney allograft loss and are considered among the most important barriers limiting long-term outcomes [8, 16, 17]. In the Banff’13 updated report [15], two principal phenotypes of acute ABMR were defined, including acute ABMR phenotype 1 in pre-sensitized patients, and acute ABMR phenotype 2, which develops with the emergence of *dn*DSA after transplant, probably resulting from nonadherence to, or excessive reductions in immunosuppressive therapies [18, 19]. Preventing the development of *dn*DSA would certainly be the most effective strategy to prevent the phenotype 2 acute ABMR, however the ideal immunosuppressive therapy to achieve this objective has not yet been defined [18]. Because acute rejection seems to be the principal risk factor for decreased allograft survival in patients with *dn*DSA [8, 20], the next urgent challenge is to preclude or limit the initiation of the *dn*DSA -mediated allograft endothelial cell injury as soon as the *dn*DSA is detected. However, accurate preemptive therapeutic strategies have not yet been defined.

Our study shows that high-dose IVIG alone was not sufficient to prevent acute ABMR, as almost 20% of the treated patients (2/11) developed acute ABMR within one year after *dn*DSA detection. In the field of kidney allograft transplantation, a number of preventive treatments have been reported for pre-sensitized patients, including single treatments or combinations of high-dose IVIG, anti-CD20 antibody, plasmapheresis, bortezomib, and monoclonal antibody to C5 [21–27]. In one randomized double-blind trial analyzing the efficacy of high-dose IVIG alone in pre-sensitized kidney allograft recipients, the IVIG-treated group had lower panel reactive antibody, higher rate of deceased-donor transplants, shorter time to transplantation and similar 2-year graft survival, albeit at the expense of higher rejection rate [23]. Treatment with IVIG and rituximab was evaluated in a randomized trial, and showed a significant decrease in acute ABMR, and improved serum creatinine at 6 and 12 months after transplant [27]. The benefit of adding plasmapheresis and rituximab to high-dose IVIG was evaluated in a retrospective study with a historical control group, and showed less microvascular inflammation and histological changes of chronic ABMR in the most intensively treated group [26]. Finally, the limited data available suggest that desensitization with high-dose IVIG alone is inferior to combined treatment with high-dose IVIG and rituximab, or rituximab and plasmapheresis, consistent with our pilot study which indicates that IVIG has only minor effects on anti-HLA antibody outcomes when used alone. So more, it could be interesting to determine high risk ABMR patients after *dn*DSA and analyze in this subgroup high dose IVIG effect.

We also found that high-dose IVIG therapy does not affect blood leukocyte phenotype in patients with *dn*DSA. Several mutually non-exclusive mechanisms have been proposed to contribute to the efficacy of IVIG therapy, including inhibition of innate and adaptive immune cell activation and synthesis of inflammatory mediators, and induction of anti-inflammatory cells and molecules [28]. Observed benefits of IVIG on B-cell-induced graft injury are potentially mediated by their large spectrum of functions, including anti-idiotypic circuits, inhibition of inflammatory cytokine generation, inhibition of complement-mediated injury, and inhibition of antibody production [29]. However, effects of IVIG on B cell phenotype and function have been only partially described. In our study, high-dose IVIG did not change the proportion of circulating T cells, B cells, NK cells or monocytes in kidney transplant patients. We also did not observe any significant increase in transitional or naïve B cells that are likely to exhibit regulatory properties, or to be associated with tolerance status in renal transplant recipients [30–32]. In contrast, it has recently been shown that IVIG could induce *in vitro* B-cell unresponsiveness similar to anergy, and the expression of FcγRIIB, an important B-cell inhibitory receptor, while also modulating CD19 expression and decreasing expression of BAFF, a master regulatory cytokine for B-cell homeostasis [33]. However, the only transcript level affected in our study was that of CD32a (FcγRIIA), which significantly decreased, rather than increased in patients treated with high-dose IVIG. The intensity of expression and activity of FcγRIIA and FcγRIIB, which are co-expressed on human innate immune cells, determine the severity of the inflammatory response to IgG immune complexes [28]. However, a potential link between the decrease in FcγRIIA mRNA expression and DSA production remains to be established since no significant in DSA was observed in the IVIG-treated group.

In conclusion, our study suggests that treatment with high-dose IVIG alone in renal transplant recipients with *dn*DSA does not result in any significant clinical or immunological benefit. Although the number of patients studied was limited, our study design included complete and repeated monitoring, and was appropriate to identify potential benefits of high-dose IVIG alone in kidney transplant recipients with *dn*DSA. It becomes urgent to better define the most appropriate use of IVIG in the therapeutic management of these patients. It has been estimated that the average cost for a single 140 g IVIG treatment in the U.S. is approximately $6500 [34]. Repeated high-dose infusions of IVIG are now broadly used in transplant patients all over the world, thereby generating a major additional cost to transplant management without first defining the best clinical indications. Large randomized clinical studies are thus needed to clarify the best therapeutic approach to prevent microvascular injury in renal transplant recipients with *dn*DSA.

## Supporting information

S1 Table Supplemental information. (DOCX)

## Abbreviations

ABMR: antibody-mediated rejection
dnDSA: *de novo* donor-specific antibodies
DSA: donor-specific antibodies
eGFR: estimated glomerular filtration rate
HLA: human leucocyte antigen
IVIG: intravenous immunoglobulin
MFI: mean fluorescence intensity
PBMC: peripheral blood mononuclear cell
SD: standard deviation
TCMR: acute T-cell mediated rejection
