# Supplementary material for: Intravenous immunoglobulin therapy in kidney transplant recipients with de novo DSA: Results of an observational study
Source: PLoS One. 2017 Jun 27;12(6):e0178572. doi: 10.1371/journal.pone.0178572 (PMC5487035; doi:10.1371/journal.pone.0178572)
Supplement: S1 Table — (DOCX) [file pone.0178572.s001.docx]

**Supplemental information**

**Table S1: DSA characteristics and chronic lesion histology at the time of the second biopsy**

| **Variables** | **IGIV+ (n=11)** | **IGIV- (n=9)** | **P** |
| --- | --- | --- | --- |
| **Delay from transplant, months, median (Range)** | 13 (12-13) | 14 (12-25) | 0.20 |
| **Delay from the first biopsy** | 9 (7-10) | 8 (6-21) | 0.89 |
| **Class I DSA** |  |  |  |
| N (%) | 2 (18) | 2 (22) | 0.37 |
| Number, median (Range) | 0.5 (0-2) | 0 (0-1) | 0.63 |
| MFI max, median (Range) | 2118 (736-7823) | 774 (545-1605) | 0.39 |
| MFI sum, median (Range) | 2118 (1027-10410) | 774 (545-3208) | 0.57 |
| **Class II DSA** |  |  |  |
| N (%) | 7 (64) | 4 (44) | 1.00 |
| Number, median (Range) | 1 (0-1) | 1 (0-1) | 0.50 |
| MFI max, median (Range) | 928 (953-1065) | 7438 (796-16197) | 0.92 |
| MFI sum, median (Range) | 1024 (802-9156) | 1601 (590-11085) | 0.91 |
| **Histology** |  |  |  |
| Glomerulitis, N (%) | 2 (18) | 0 (0) | 0.49 |
| Grade, 1/2/3 | 1/1/0 | . | . |
| Peri-tubular capillaritis, N (%) | 1 (9) | 1 (11) | 1.00 |
| Grade, 1/2/3 | 1/0/0 | 1/0/0 | . |
| Interstitial inflammation, N (%) | 0 (0) | 0 (0) | . |
| Tubulitis, N (%) | 1 (9) | 1 (14) | 1.00 |
| Grade, 1/2/3 | 1/0/0 | 1/0/0 | . |
| Chronic glomerulopathy, N (%) | 1 (9) | 0 (0) | . |
| Grade, 1/2/3 | 1/0/0 | . | . |
| Interstitial fibrosis, N (%) | 6 (55) | 5 (55) | 1.00 |
| Grade, 1/2/3 | 4/1/1 | 3/2/0 | 0.72 |
| Tubular atrophy, N (%) | 6 (55) | 5 (55) | 1.00 |
| Grade, 1/2/3 | 5/0/1 | 4/1/0 | 0.70 |
| Vascular |  |  |  |
| cv | 3 (27) | 5 (71) | 0.14 |
| Grade, 1/2/3 | 2/1/0 | 3/2/0 | 0.12 |
| ah | 7 (64) | 4 (57) | 0.64 |
| Grade, 1/2/3 | 6/1/0 | 1/3/0 | 0.71 |
| C4d+, N (%) | 0 (0) | 0 (0) | . |
| **Acute rejection** |  |  |  |
| Antibody-mediated, N (%) | 2 (18) | 0 (0) | 0.46 |
| T-cell mediated, N (%) | 0 (0) | 0 (0) | . |
| Mixed, N (%) | 0 (0) | 1 (11) | 1.00 |
| **Delay, months, median (range)** | 10 (9-12) | 14 | . |
|  |  |  |  |
| **eGFR (ml/min/1,73m2), mean (SD)** | 57 (±18) | 54 (±17) | 0.72 |
| **Proteinuria (g/L), median (range)** | 0.09 (0.07-0.30) | 0.08 (0.05-0.15) | 0.46 |
